# Pain processing in primary headaches, from spinal sensitisation to cortical modulation

**DOI:** 10.1186/1129-2377-16-S1-A12

**Published:** 2015-09-28

**Authors:** Armando Perrotta

**Affiliations:** IRCCS Neuromed, Pozzilli, IS Italy

In primary headaches, such as migraine and cluster headache, an abnormal processing of pain stimuli at trigeminal, as well as at spinal level, has been demonstrated. As in other pain conditions, also primary headaches are accompanied by central sensitisation of pain pathways at cephalic and extra-cephalic level. Interestingly, in primary headaches the facilitation in pain processing has been detected during either the attack or active phase, as well as during the interictal period[1–3]. The interictal facilitation in pain processing in patients with episodic migraine or cluster headache during the active phase but outside the attack, reflects a generalised defective function of the pain system, probably as a consequence of a genetic substrate. This chronic hyperexcitability may contribute to their susceptibility to develop recurrent pain attacks. In the chronic form of migraine, as well as in cluster headache during the active phase of the disease, the hypersensitivity of the pain system is related to a dysfunction in central supraspinal antinociceptive pathways modulating pain processing[2, 4, 5]. In migraineurs, a high frequency of migraine attacks coupled with an overexposure to symptomatic medication contributes to a further impairment in nociceptive control, leading to the progression from episodic to chronic form of migraine and medication-overuse headache. This hypothesis is supported by a recovery to a normal functioning of both the supraspinal antinociceptive system and pain sensitivity after withdrawal treatment or clinical improvement. In cluster headache, the state-dependent facilitation in pain processing is linked to a state-dependent defective supraspinal control of pain, which is normally operating during the remission phase of the disease. On these bases, an imbalance of excitatory and inhibitory systems supports the development of episodic, remittent and chronic pain conditions in subjects with a pro-nociceptive profile. Interestingly, a series of studies demonstrated morphologic and metabolic abnormalities in pain-related brain areas in subjects with chronic form of migraine and cluster headache. Our recent data have demonstrated that the facilitation in pain processing and the related defective supraspinal control of pain are linked to a dismodulation of the default mode network.
